# Evaluation of Root-End Preparation with Two Different Endodontic Microsurgery Ultrasonic Tips

**DOI:** 10.3390/biomedicines8100383

**Published:** 2020-09-28

**Authors:** Paulo J. Palma, Joana A. Marques, Margarida Casau, André Santos, Francisco Caramelo, Rui I. Falacho, João Miguel Santos

**Affiliations:** 1 Institute of Endodontics, Faculty of Medicine, University of Coimbra, 3000-075 Coimbra, Portugal; joanaamarques@hotmail.com (J.A.M.); jsantos@fmed.uc.pt (J.M.S.); 2 Center for Innovation and Research in Oral Sciences (CIROS), Faculty of Medicine, University of Coimbra, 3000-075 Coimbra, Portugal; 3 Dentistry Department, Faculty of Medicine, University of Coimbra, 3000-075 Coimbra, Portugal; amcasau@hotmail.com (M.C.); vazsantos.andre@gmail.com (A.S.); 4 Coimbra Institute for Clinical and Biomedical Research (iCBR), Laboratory of Biostatistics and Medical Informatics (LBIM), Faculty of Medicine, University of Coimbra, 3000-548 Coimbra, Portugal; fcaramelo@fmed.uc.pt; 5 Institute of Oral Implantology and Prosthodontics, Faculty of Medicine, University of Coimbra, 3000-075 Coimbra, Portugal; rifalacho@fmed.uc.pt

**Keywords:** apical surgery, endodontic microsurgery, root-end preparation, ultrasonic, ultrasonic tips

## Abstract

The aim of this study is to compare root-end preparation performed with two different ultrasonic tips—CVDentus and NSK—and respective time requirements. After root-end resection, 32 teeth were randomly divided in two groups, according to the ultrasonic tip used for root-end preparation. Preparation time was recorded. Photomicrographs were taken to assess the following parameters: root surface microcracking, marginal integrity and presence of debris. One ultrasonic tip from each group was analyzed through scanning electron microscopy before and after root-end preparation. The significance level was set at α = 0.05. Incidence of microcracks in both groups was 12.5%. Solely intracanal microcracking was found, consistently positioned within the widest side of the remaining dentine. No statistically significant differences were verified between both experimental groups regarding marginal integrity (*p* = 0.102) and preparation time (*p* = 0.780), whereas statistical differences (*p* = 0.003) were found concerning the presence of debris (the minimum registered score was mostly verified in CVDentus group). NSK tips showed major morphological changes, with extensive surface wear and noticeable loss of particles, which was not verified on CVDentus tips. Our findings suggest significant differences regarding root-end preparation walls quality, with CVDentus tips showing better results. Concerning microcracking, as well as preparation time and marginal integrity, both ultrasonic tips showed similar results. Qualitative analysis exposed NSK tips major morphological changes and wear after use, which was not verified on CVDentus tips.

## 1. Introduction

Apical periodontitis comprises the host’s response to pathogenic microorganisms colonizing the root canal system of the tooth [1,2]. The principal goal of conventional endodontic treatment is prevention and/or elimination of apical periodontitis [1,2]. Today, the success rate of endodontic treatment stands between 85% and 95%, being frequently applied to treat irreversible inflammation or necrosis of the root canal content [3]. Endodontic microsurgery is often the last option when nonsurgical retreatment fails, is unfeasible or unlikely to improve the initial endodontic treatment [4]. In particular, only surgical intervention may resolve cases involving a persistent lesion with etiology related to complex canal anatomy, extra-radicular infection, foreign body reaction material, and/or cystic tissue [3].

Periapical surgery facilitates complete debridement of the root canal and placement of a root-end filling to ensure adequate apical sealing. The surgical approach comprises several sequential procedures in order to fulfill the aforementioned goals: (a) periapical resection (apicoectomy), (b) preparation of the root-end cavity, and (c) sealing of the root canal system by means of a bioactive and biocompatible root-end filling material placement [4,5].

The advent of novel diagnostic tools, instruments, and materials has greatly benefited endodontic surgery [5,6]. Forty-five-degree root-end resection bevels, bur driven retrograde preparations and amalgam or intermediate restorative materials for root-end filling were for many years considered the state-of-the-art with inconsistent success rates ranging from 44.2% to 59% reported prior to the introduction of microsurgical techniques [7,8].

The introduction of the dental operative microscope (DOM) in the early 1990s led to a new era in modern microsurgical endodontics [9]. Besides magnification, contemporary techniques incorporate the use of ultrasonic tips, microsurgical instruments and filling materials which exhibit superior biocompatibility such as mineral trioxide aggregate (MTA), Biodentine [7,10,11,12,13], and premixed tricalcium silicate putty (TotalFill FS putty) [14]. This new microsurgical approach allowed for a significant improvement in success rates, reaching levels above 91% [7,15].

The available literature highlights the importance of an adequate root-end preparation for a favorable prognosis, with its quality being directly related to the treatment success [16]. Root-end preparation should be parallel to the long axis of the root, 3 mm deep, and centered within the root in order to preserve adequate wall thickness and retain a biocompatible filling material [4,5,17]. 

Ultrasonic tips present an alternative to the conventional rotary burs and show several advantages when used to perform root-end preparation. In fact, the advent of ultrasonic tips resulted in the improvement of root-end preparation mainly due to the availability of tips with different shapes and angulations, which are meticulously selected according to the root features and location [18]. Moreover, ultrasonic tips carry numerous advantages including the possibility of performing a more conservative osteotomy and of obtaining root-end resection with minimal or inexistent bevel angles [19], thus reducing the number of exposed dentinal tubules and consequently the possibility of apical leakage [20]. Additionally, these tips enable the removal of isthmus tissue present between two canals within the same root [5] and exhibit lower risk of damaging the surrounding soft tissues during the surgical procedure [7]. Ultimately, ultrasonic preparation results in root-end cavities that are smaller, cleaner, and more retentive, as well as more centrally placed and aligned with the direction of the original root canal [17]. However, the incidence of apical microcracks following root-end preparation with ultrasonic tips has been reported [21,22,23,24]. 

Although not formally established, microcracks may increase the chance for apical leakage and jeopardize the overall strength of the root-end [25], with negative influence in the long-term outcome of endodontic microsurgery [10]. Optical magnification with or without the use of dyes [9,26], histological sections [26], stereomicroscopy [27], scanning electron microscopy (SEM) [28], and fluorescence confocal microscopy [26] are commonly used methods for detecting microcracks resulting from ultrasound-activated root preparation. A few factors have been identified that potentially contribute to the occurrence of microcracks—namely, the use of dehydrated extracted teeth, absence of periodontal ligament, improper power settings of the ultrasound unit, sputter-coating of specimens for SEM examination, time required for root-end preparation, initial root condition, and remaining dentinal thickness [29]. Additionally, the type of coating of the ultrasonic tips may play a significant role in microcrack development [30].

Recently some attempts have been made to improve ultrasonic instruments both in terms of usability, as well as performance. New zirconium-coated and diamond-coated root-end preparation tips represent a relevant issue in this field [31]. However new technologies arise, such as the chemical vapor deposition which comprises the formation of a thick pure diamond layer that shall produce a single stone covering the entire tip’s surface [32].

The aim of the present ex vivo study is to compare root-end preparation performed with two different ultrasonic tips—chemical vapor deposition CVDentus (CVDentus, São Paulo, Brazil) and diamond-coated NSK (NSK, Tochigi, Japan)—regarding root surface microcracking (throughout the seven-day evaluation period), quality of the root-end cavity margins, presence of debris, root-end preparation tips’ wear, and respective time requirements.

The null hypothesis states there are no differences between both ultrasonic tips regarding the evaluated parameters.

## 2. Materials and Methods

### 2.1. Specimen Selection

The present study has been approved by the Ethical Committee of the Faculty of Medicine of the University of Coimbra (notification CE001/2013, 2 February 2015) and followed the guidelines of the Declaration of Helsinki. Forty single-rooted premolars with fully developed apices, freshly extracted for orthodontic reasons, were selected. Sample size calculation was based on a previous, unpublished, pilot study using G* Power (3.1.9.3 software, Kiel, Germany), considering a significance level of 5% and a power of 80%. All teeth were immersed in 1% sodium hypochlorite (NaOCl, CanalPro, Coltene/Whaledent AG, Altstatten, Switzerland) for 15 min, immediately after extraction. Afterward, soft tissue and debris were removed from the external root surfaces with periodontal scalers. The integrity of the roots was assessed using DOM (Leica M300 Surgical microscope, Leica Microsystems, Wetzlar, Germany) under 16× magnification. Teeth were then kept immersed in 0.5% chloramine T for a period of one to three weeks in an incubator (Gallenkamp, London, United Kingdom) with controlled temperature of 37 °C, to simulate the oral environment clinical conditions.

### 2.2. Specimen Preparation

Teeth were decoronated using a high-speed conical trunk diamond bur (Infinity, CVDentus, São Paulo, Brazil) under continuous water spray. The working length was determined using a size 10 K-file (a 0.5 mm distance from the apex was considered as reference for working length determination). Root canals were then cleaned and mechanically prepared up to F2 (ProTaper universal, Dentsply Maillefer, Baillaigues, Switzerland) applying a crown-down technique. The root canals were irrigated with 1 mL of 1% NaOCl between each file usage, totaling a volume of irrigant solution of 4 mL. When preparation was completed, a final rinse with 2 mL of 70% alcohol (Meda Pharma, Lisboa, Portugal) was performed, and root canal system was then dried using sterile absorbent paper points (Zipperer Absorbent Paper Points Endo Easy Efficient, VDW; Munich, Germany).

The single cone technique was then used for root canal filling with calibrated gutta-percha ProTaper points F2 (ProTaper universal, Dentsply Maillefer, Baillaigues, Switzerland) and AH Plus (Dentsply, Konstanz, Germany) as sealer. Section of the gutta-percha cone was performed at the cement enamel junction (CEJ) by using a heated instrument and then vertically condensed with Buchanan System B Pluggers (SybronEndo, Orange, CA, USA).

Following obturation, each tooth was numbered, and an X-ray image was taken to confirm the quality of obturation. Finally, teeth were positioned prior to the subsequent procedures by placing the root’s two coronal thirds in high—viscosity silicone material (Coltène Lab-Putty, Coltène/Whaledent AG, Switzerland). 

All specimens were stored in an incubator (Gallenkamp, London, UK) at 37 °C and 98% humidity throughout the experimental period.

### 2.3. Root-End Resection

The section level was set at 3 mm from the apex, and all the roots were resected according to a 90-degree angle to their longitudinal axis. Root-end resection was performed using a H23LR (Komet, Gebr. Brasseler, Lemgo, Germany) carbide tungsten operative bur and the section surface was posteriorly smoothed with a H375R (Komet, Gebr. Brasseler, Lemgo, Germany) carbide tungsten finishing bur.

Hereafter, the root surfaces were checked by an examiner, with a DOM (Leica M300 Surgical microscope, Leica Microsystems, Wetzlar, Germany) under 16× magnification, to assess the presence of microcracks. Photomicrographs of the cutting section were taken with a stereomicroscope (objective HR Plan Apo 1X WD 54—Nikon SMZ 1500, Tokyo, Japan) before and after methylene blue dye 1% (Canal blue, DentsplySirona, Konstanz, Germany), which was applied directly on the surface during 5 min, and followed by rinsing with abundant water for 1 min, in order to improve microcracks visualization.

### 2.4. Root-End Preparation

Thirty-two teeth that did not present any microcracks or fractures were stratified by transversal root shape and surface area, and randomly divided in two groups (stratified random sampling method), according to the ultrasonic tip used in root-end preparation:

- Group 1 (n = 16): ultrasonic chemical vapor deposition tip TOF-L (CVDentus, São Paulo, Brazil)—lot number E7009;
- Group 2 (n = 16): ultrasonic diamond-coated tip E32D (NSK, Tochigi, Japan)—lot number Z217211.

Root-end preparation was performed using the matching ultrasound unit and following the manufacturers’ recommendations regarding intensity, namely 30% intensity of power when using CVDentus Ultrasonic System (CVDentus; São Paulo, Brazil) and Endo mode level 6 of intensity with Varios 970 (NSK iPiezo engine, Tochigi, Japan), under continuous saline solution irrigation. Root-end preparation was performed applying intermittent and minimal pressure, with in-and-out motion until an apical cavity 3 mm deep from the resected surface was achieved, followed by circumferential movements to complete the entire preparation. Specimens were kept in the silicone blocks and maintained hydrated throughout the procedures. Each tip was used on a maximum of eight roots and replaced in case of tip fracture. This procedure was accomplished by a single operator, using a DOM (Leica M300 Surgical microscope, Leica Microsystems, Wetzlar, Germany) under 16× magnification. The root-end cavity was considered finished when the operator deemed to have obtained a visibly debris-free preparation. All preparations were class I (according to Black’s classification). Preparations were recorded using a video camera and time was measured using a video playback software in order to get a more precise measurement, counting solely the actual time of tip-root-end contact.

Photomicrographs were taken following preparation of each root before and immediately after (T_PO_) applying methylene blue dye 1% (Canal blue, DentsplySirona, Konstanz, Germany) as previously described, as well as 24 h (T_24H_) and seven days (T_7D_) after root-end preparation.

### 2.5. Data Analysis

The preoperative and the postoperative photomicrographs were coded and evaluated by two blinded operators. The examiners assessed the following criteria, through photomicrographs analysis under 20× and 40× magnification:

- The number, type and location (in relation to dentinal walls) of root surface microcracking (Table 1a);
- The quality of root-end cavity margins produced by the ultrasonic tips (Table 1b).

Additionally, direct stereomicroscope visualization under 40× magnification allowed for the evaluation of the presence of debris (dentinal and/or gutta-percha remnants)—Table 1c.

The scores and number of cracks were assessed independently by two examiners, and in case of disagreement both examiners discussed until a consensus was reached.

In addition, one ultrasonic tip from each experimental group was randomly selected and analyzed through scanning electron microscopy (SEM) before and after root-end preparation, with the purpose of evaluating tip wear due to use.

### 2.6. Statistical Analysis

Statistical analysis was carried out using the commercially available IBM SPSS v.24 software (Chicago, IL, USA) to assess the differences between the experimental groups. In order to evaluate the incidence of microcracks before and after (T_PO_, T_24H_, T_7D_) root-end preparation, as well as cracking type and location, results obtained for each group were analyzed through descriptive statistics. The Mann—Whitney test was performed to evaluate the differences regarding microcracks, marginal integrity (quality of apical cavity margins) and presence of debris between groups. Concerning the time required for root-end preparation, the normality of data distribution testing was carried out using the Shapiro–Wilk test. The Mann—Whitney test was used to detect significant differences between the groups as data did not follow the normal distribution. The significance level was set at α = 0.05.

## 3. Results

### 3.1. Root-End Surface Microcracking—Number, Type, and Location

**Figure 1:**
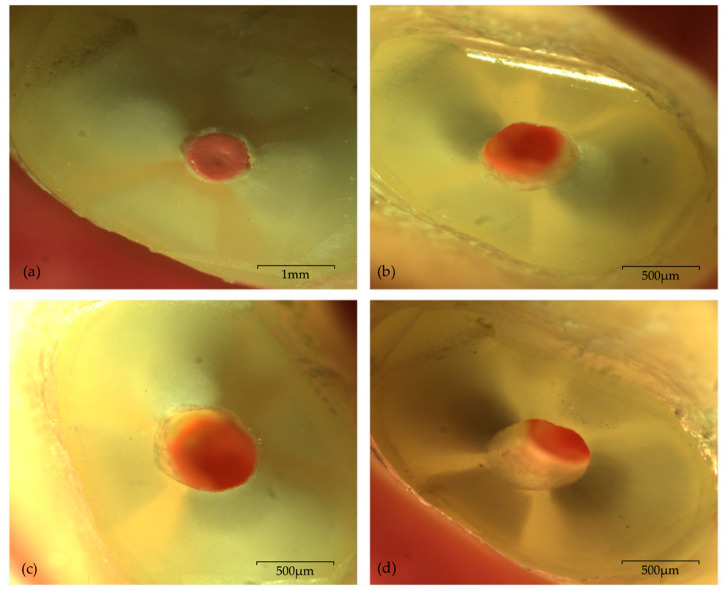
Photomicrographs of one sample from CVDentus group with no visible microcrack, under 20× magnification: (**a**) immediately after root-end resection; (**b**) immediately after root-end preparation—T_PO_; (**c**) 24 h after root-end preparation—T_24H_; (**d**) seven days after root end preparation—T_7D_.

**Figure 2:**
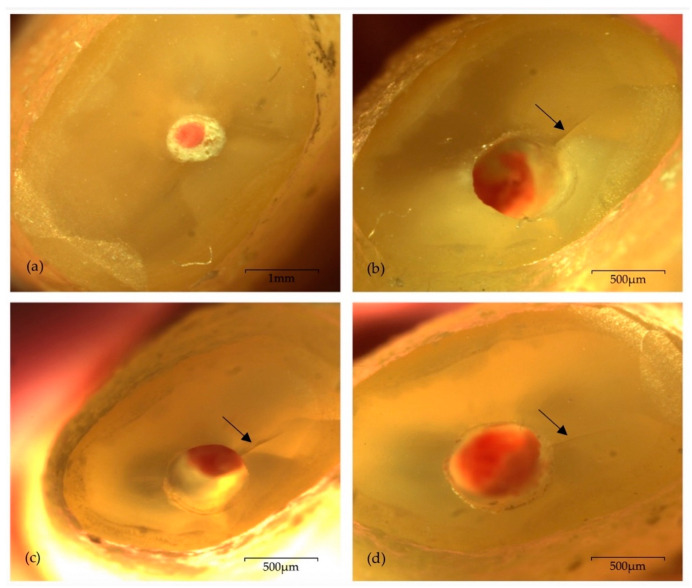
Photomicrographs of one sample from NSK group presenting one intracanal microcrack (arrow) located within the “wider” part of the remaining dentine walls, under 20× magnification: (**a**) immediately after root-end resection; (**b**) immediately after root-end preparation—T_PO_; (**c**) 24 h after root-end preparation—T_24H_; (**d**) seven days after root-end preparation—T_7D_.

Table 2 shows the results of the two study groups regarding the number, type and location of cracks. No visible cracks were detected after root-end resection, independently of the tip type. Regardless of the timepoint (T_PO_, T_24H_, T_7D_) following root-end preparation, intracanal root microcracking was observed in two samples of each experimental group. Therefore, an incidence of 12.5% was recorded concerning the occurrence of fractures in both groups. No propagation of fractures, nor the appearance of new ones, was verified throughout the complete seven-day period of evaluation. Moreover, the maximum number of microcracks recorded for the same sample was one. No extra-canal, intra-dentine, or complete microcracks were found. Regarding location, root surface microcracking was consistently positioned within the widest side of the remaining dentine surface, thus registering a frequency of 100% for the “wider” part of the root location. Figure 1 (CVDentus group) and Figure 2 (NSK group) display representative images of root surface microcracking of both experimental groups.

### 3.2. Marginal Integrity (Root-End Cavity Margins)

**Figure 3:**
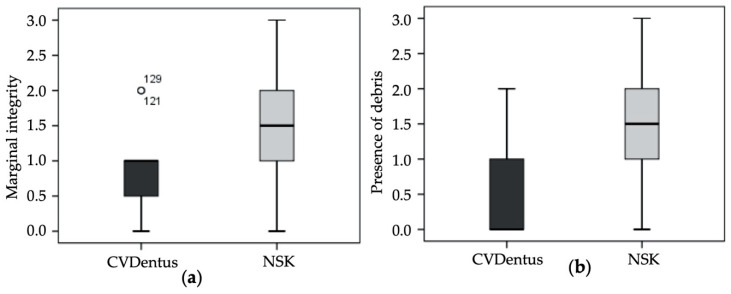
Score distribution within the tested groups regarding (**a**) marginal integrity (Mann—Whitney test; *p* = 0.102) and (**b**) presence of debris. (Mann—Whitney test; *p* = 0.003).

Regarding marginal integrity (Table 3a), the maximum value of “3” was found in one root from NSK group, totaling 6.2% of the samples from the referred experimental group. The minimum registered score was “0” mostly verified in specimens from CVDentus group (25% of the samples from the group). The score “1” was the one with highest incidence in CVDentus group with a percentage of 62.5%, whereas in NSK group the value with the highest incidence was “2” (43.8%). No statistically significant differences (U = 84.00; Z = −1.783; *p* = 0.102) were verified between both CVDentus and NSK groups regarding marginal integrity (Figure 3a).

### 3.3. Presence of Debris (Walls Quality)

**Figure 4:**
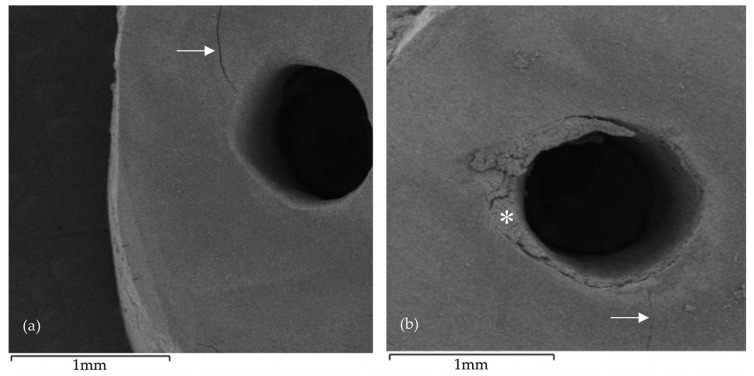
Scanning electron microscopy (SEM) images following root-end preparation: (**a**) CVDentus specimen exhibiting an intracanal microcrack (arrow); (**b**) NSK specimen exhibiting an intracanal microcrack (arrow) and irregular root-end cavity margins, as well as showing visible accumulation of debris (asterisk) resulting from the root-end preparation.

Concerning the presence of debris following root-end preparation (Table 3b), the maximum value of “3” was found in one sample from NSK group (6.2% of the samples from the referred experimental group). The minimum registered score was “0” mostly verified in specimens from CVDentus group (56.2% of the samples from the group). In NSK group the score exhibiting the highest incidence was “2” with a percentage of 43.8%. Contrariwise to marginal integrity, statistically significant differences (U = 50.50; Z = −3.093; *p* = 0.003) were found between the tested groups regarding the presence of debris (Figure 3b and Figure 4).

### 3.4. Time Requirements

Considering the time requirements to perform apical preparation (Table 4), no statistically significant differences could be detected between the tested groups (U = 120.00; Z = −0.302; *p* = 0.780).

### 3.5. Root-End Preparation Tip Wear—Qualitative Analysis

**Figure 5:**
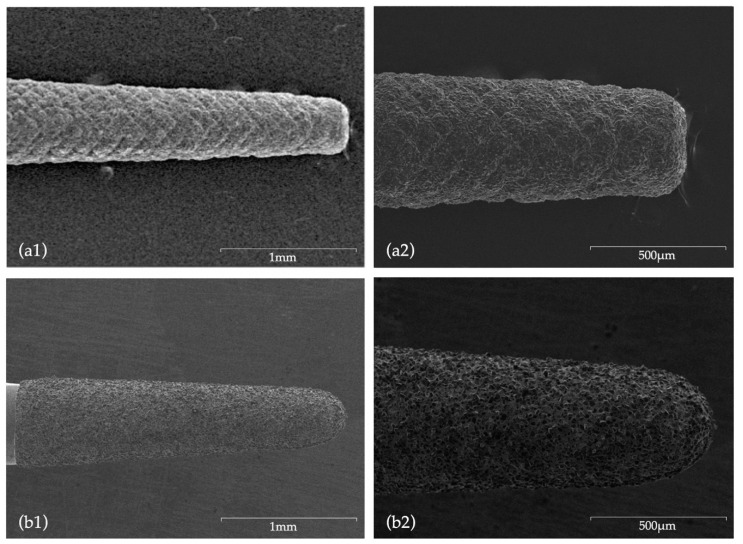
SEM images before use: (**a1**) CVDentus tip (50× magnification); (**a2**) CVDentus tip (100× magnification); (**b1**) NSK tip (50× magnification); (**b2**) NSK tip (100× magnification).

**Figure 6:**
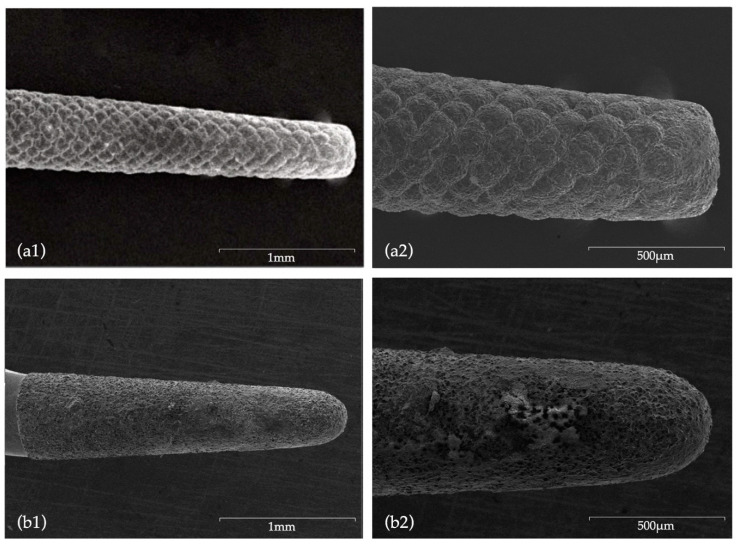
SEM images after use: (**a1**) CVDentus tip (50× magnification); (**a2**) CVDentus tip (100× magnification); (**b1**) NSK tip (50× magnification); (**b2**) NSK tip (100× magnification).

Figure 5 and Figure 6 depict images of the ultrasonic tips obtained by scanning electron microscopy. (SEM) examination, showing tip wear after root-end preparation. No ultrasonic tip fractures were registered in any of the groups.

## 4. Discussion

Since the introduction of ultrasound technology for root-end preparation in the 1990s, several studies have confirmed it as a technical improvement with positive impact in the clinical outcome of endodontic microsurgery [4,15,35,36,37]. Hence, the present study contributes with uttermost relevance to the improvement of a technique that has proven to greatly affect the treatment outcome and tooth survivability, as well as to understanding which materials may perform better in root-end preparation and consequently provide conditions for long-term clinical success with less debris and possible improvement of the root-end seal which may prevent recurring pathology. The aim of this study was to evaluate time requirements and compare the features of root-end preparation performed with two different ultrasonic tips regarding root surface microcracking, marginal integrity, and presence of debris, as well as evaluate tips’ wear.

Adequate root-end preparation is an important step for treatment success [38]. The increased cavity depth achieved with ultrasonic tips might be a significant factor for apical microleakage control [19]. Furthermore, higher clinical success rates were reported when root-end preparation was performed using ultrasonic tips rather than classic rotary burs approach [7,11,12,13,18]. The specific use of ultrasonic devices in endodontic microsurgery of molars is highlighted in a prospective randomized study presenting a significantly higher success rate in comparison with conventional techniques, which might be related to the easier access inherent to ultrasonic tips [18].

### 4.1. Root Surface Microcracking

The formation of microcracks following root-end resection or root-end preparation presents a clinical concern [28]. Ultimately, root-end microcracking might result in increased susceptibility to root fracture, inability to properly seal the apical preparation, and the possibility of additional bacterial contamination and apical leakage, therefore impairing the outcome of the treatment [31,34,39]. Although some studies identify microcracking as the main inconvenience of the use of ultrasounds in apical preparations [30,40], other published results indicate disagreement within literature on this topic [41,42].

Nevertheless, aiming at an upgrade of ultrasonic results in regard to microcrack formation following apical preparation, new diamond-coated tips have been introduced [43,44]. The production of novel diamond-coated ultrasonic tips, such as CVDentus (CVD) tips, was achieved with the objective of obtaining tips with greater cutting efficiency, which therefore would require lower preparation time, thus possibly reducing the incidence of fractures [32,43,44,45]. Previous studies already confirmed that CVD tips present higher cutting effectiveness when compared to conventional ultrasonic diamond instruments, as well as a shorter root-end preparation time [32,45]. Camargo Villela Berbert et al. [46] used CVD tips in apical preparation without any verified resultant root-end surface damage, thus corroborating these results.

#### 4.1.1. Preparation Time

The time required for root-end preparation presents a relevant clinical parameter which must be taken into high consideration [17,22,28,47,48]. Khabbaz et al. [34] found that the time needed to prepare a root-end cavity with conventional rotary technique was shorter when compared to sonic/ultrasonic tips, which is in agreement with the results of Waplington et al. [49], whereas Engel and Steiman [42] reported similar preparation time when comparing rotary burs with smooth ultrasonic tips. However, in clinical context, ultrasonic instruments may be faster since they allow better and easier accessibility to the root-end and require less bone removal. Furthermore, Taschieri et al. [31] and Peters et al. [48] observed that root-end preparation with diamond-coated tips is significantly faster than with the stainless-steel ones. In addition, Peters et al. [48] found a correlation between the incidence of microcracks and the time needed to accomplish root-end preparation, which was lately confirmed by Tobon-Arroyave et al. [22], being a higher preparation time associated with microcrack formation. Also, published data establishes that the quality of the preparation is directly related to the preparation time, with the preparations performed in less time showing superior results [28,31]. In the present study no statistically significant differences were detected in preparation time between the tested groups, with CVD group exhibiting a mean preparation time of 60.56 s, while NSK group presented a mean value of 55.56 s. The time requirements recorded in this study were higher than those verified by Bernardes et al. [32], who registered the following mean times for apical cavity preparation: 17.94 s (CVD), 44.83 s (Trinity), and 45.57 s (Satelec).

#### 4.1.2. Incidence, Type, and Location of Microcracking

Primarily, regarding preoperative cracking, Onnink et al. [50] reported significant differences between instrumented vs non-instrumented root canals. Therefore, in this experimental work, only teeth which did not present any cracks or fractures following root-end resection were included, similarly to Liu et al. [28,51,52] experimental protocol. The incidence of microcracks in the present study was 12.5% for both groups, corresponding to two cracks within each group, therefore no statistically significant differences were verified between the two tested tips regarding this parameter. Despite being in agreement with Liu et al. [28,51,52], our results differ from Bernardes et al. [32], in which no microcracks or fractures were reported following root-end preparation with three different diamond tips (including CVD tips), likewise by Batista de Faria-Junior et al. [53]. Khabbaz et al. [34] also did not find any microcracks after root-end cavity preparation with sonic and ultrasonic diamond tips. In contrast, Peters et al. [48] obtained an incidence of cracks of 2.1% and 4.7%, respectively.

Moreover, the sequential analysis of photomicrographs taken at T_PO_, T_24H_ and T_7D_ revealed that there was no propagation of microcracks, nor the appearance of new ones over time. However, it is noteworthy that the evaluation period (seven days) may have been short to assess the effect of microcrack propagation. Further studies, with longer “follow-up” period, are needed to verify this hypothesis. Tawil [54] provided data on the effect of ultrasonic root-end preparation on dentinal defect creation and propagation, having concluded that ultrasonic root-end preparations are safe to use on intact roots, but also that preexisting dentinal defects can be propagated by the ultrasonic approach.

In the present study, regardless of the evaluation period following root-end preparation, only intracanal root microcracking was observed, thus no statistically significant differences were verified between the experimental groups regarding the type of root surface cracking. These results are in agreement with the study of Taschieri et al. [31].

Regarding location, root surface cracking was consistently positioned within the widest side of the remaining dentine, similarly to the results obtained by De Bruyne and De Moor [33], in which only few microcracks were located within the narrower dentine walls. These results contrast with those of former studies in which most microcracks developed in the thinnest walls surrounding the root-end cavity preparations [30] or small diameter roots developed more microcracks [55].

#### 4.1.3. Potential Protocol Variables

##### Experimental Model

Literature suggests some of the in vitro conditions in which several studies were performed as a possible reason for the formation of microcracks: stresses exerted during extraction (either traumatic or atraumatic extraction techniques) [56,57], possible tooth dehydration, inappropriate storage and careless handling of the extracted teeth, as well as absence of periodontal support [27,29]. Therefore, in the present ex vivo study we could have obtained an overestimation of microcracks, despite all the efforts that have been made to prevent it—only freshly extracted teeth were included and specimens were kept moist throughout the experimental procedures, along with the use of silicon blocks to minimize the concern of the absence of periodontal ligament and stabilize the teeth during instrumentation procedures, although in Gondim et al. [58], the use of stabilization methods did not prevent the appearance of fractures after root-end preparation. In order to avoid artifacts and obtain results which are more clinically relevant, some authors claim that investigations should be preferably performed in situ [25,27,33]. Calzonetti et al. [27] suggested that, in situ, roots may absorb some of the ultrasonic impact and prevent microcracks propagation, overcoming tooth dehydration and brittleness associated with in vitro context, thus reducing the chance of artifacts. Previous studies indicate the use of cadavers as a potential suitable alternative [25,27,33,57]. Future in situ (and mostly clinical) studies are needed.

##### Power Settings

Several other factors have been outlined aiming to explain results regarding microcrack formation. Previous studies reported that varying power intensity of the ultrasound unit does not significantly influence the outcome of root-end preparations [23,25,31]. On the contrary, other results [31,40,55] show increased occurrence of microcracks when ultrasounds were used in both high-power configurations [31] or low power settings [33], depending on the considered study. Therefore, further research is required to determine the optimal power for root-end preparation. In the present study the intensity of the power setting was selected according to the value recommended by the manufacturer.

##### Magnification

Photomicrographs were taken at three different timepoints (T_PO_, T_24H_, and T_7D_) to check possible propagation of microcracks detected immediately after root-end preparation (T_PO_), as well as to identify newly formed ones. The photomicrographs were taken under 20× and 40× magnifications which, according to some studies, may have prevented the complete detection of existing microcracks, thus suggesting that the incidence of fractures may vary depending on the magnification degree [33,49,58,59,60,61]. Hence, magnification arises as a factor to be considered in the detection of microcracks.

### 4.2. Marginal Integrity

Apart from microcracks, chipping was also referred as a consequence of ultrasonic or sonic root-end preparation [33,58,59]. The importance of irregular margins is unclear, but the hypothesis that chipped margins may jeopardize apical sealing provided by root-end filling materials has been raised [31]. Our results regarding marginal integrity of the prepared root-end cavity indicate that both experimental groups show apical cavities with irregular margins, with the majority of the preparations having at least one defect, which agrees with the results of Khabbaz et al. [34]. In addition, no significant differences were detected between the tested tips, which is in accordance with Bernardes et al. [32].

### 4.3. Presence of Debris

In the present study, similarly to other published data, teeth were instrumented and filled so that, besides microcrack assessment, an evaluation of debris presence (remnants of gutta-percha) on the cavity walls would also be possible, thus more faithfully reproducing a clinical situation [23,31,34]. This is a crucial parameter in apical preparation and was quantified in the present study regarding the number of debris (dentin and/or gutta-percha remnants) present inside the apical cavity following preparation. CVD group was significantly better compared to NSK group, with the first presenting a considerably smaller number of debris on the preparation walls. Therefore, although the effects of debris on the treatment outcome are still unknown and require further clinical studies, CVD tips allow for higher quality root-end cavity walls. The null hypothesis is then rejected.

In addition, the difference observed in relation to the presence of debris may also have a key impact on the prognosis as it interferes with the adhesion of calcium silicate-based cements to the dental walls. Vivan et al. [62] found highest bond strength values when preparing with CVD tips, irrespective of the root-end filling material used, meaning that root-end preparation with the CVD tips positively influences the bond strength of root-end filling materials.

### 4.4. Root-End Preparation Tips Wear

Concerning tip wear, the direct comparison of SEM images of new ultrasonic tips with the same tips after being used, allowed for a wear and tear evaluation. Tips from CVD group did not present major surface changes, with no visible loss of particles, with even the active point still presenting a morphology similar to the original one. According to Bernardes et al. [32], this is attributed to the CVD manufacturing process which involved a chemical vapor diamond deposition on a molybdenum shaft exhibiting high adhesion characteristics of the diamond coating [32,53,63], ultimately ensuring a superior cutting efficiency when compared to conventional ultrasonic diamond tips [32,45]. Bernardes et al. [32] registered diamond particles loss after use in conventional ultrasonic tips formed by diamond crystals embedded in a joining material (Satelec and Trinity tips), an occurrence which was not visible in CVD tips. Contrarily, in the present study, NSK tips showed extensive surface wear, with major changes being observed in morphology and a very noticeable loss of particles not exclusively located in the active area.

### 4.5. Final Summary

Considering that the study was carried out in an ex vivo context, results regarding microcrack formation may be an overestimation in relation to the clinical reality. However, marginal integrity and the presence of debris are directly transposable to the clinic context, generating consisting meaningful results which interpretation may reveal crucial translational data.

Notwithstanding the discussion of the results presented, it is important to underline the availability of several studies exhibiting distinct experimental designs, thus making comparison difficult to achieve. In fact, the use of different types of root-end preparation tips design, materials and methods of evaluation constitute possible sources of variability [31]. Different apical diameter of the specimens included in the studies could also lead to increased outcome variability [31]. Standardization of the experimental study design is required, otherwise the comparison between different studies may lead to conclusions of limited validity.

Additionally, it is noteworthy that clinicians should be aware that root canal anatomical variations may play a major role in the success of endodontic microsurgery and that each case should be evaluated with the aid of three-dimensional imaging, namely cone beam computed tomography, which allows for both a qualitative and quantitative assessment of root canal system anatomy complexity [64,65,66]. Furthermore, nowadays new digital dentistry techniques are being developed, such as the use of virtual reality and other computer aided procedures, that might also present an effective tool to guide root-end resection and preparation [66].

Furthermore, although the present study focuses on a technical improvement of the root-end preparation, it is important to bear in mind that the success of endodontic microsurgery requires the creation of a favorable apical microenvironment that allows further healing of periapical tissues, which implies the recruitment of growth factors and cellular differentiation [67,68,69]. Thus, after preparing the apical cavity, root-end filling should include the use of a biocompatible and bioactive material, such as MTA or Biodentine, which promotes the repair/regeneration of the apical tissues [68,70]. The technical principles of endodontic microsurgery are therefore underlying a whole biological approach and new promising potential applications of regenerative strategies should be considered [71,72,73,74,75,76].

## 5. Conclusions

Within the limitations of the present study, our findings suggest significant differences between the two tested ultrasonic tips in regard to the quality of root-end preparation walls, with CVDentus tips showing better results. Concerning the number, type and location of microcracks, as well as the mean preparation time and marginal integrity, both ultrasonic tips showed similar results. The time elapsed following apical preparation itself might not be a key factor for the appearance of new cracks or propagation of existing ones. Qualitative analysis of tip wear through SEM images exposed NSK tips major morphological changes and wear after use, which was not verified on CVDentus tips.

## Figures and Tables

**Table 1:** Classifications adapted to evaluate (**a**) root surface microcracking, (**b**) quality of root-end cavity margins, and (**c**) presence of debris within the apical cavity, following root-end preparation.

(a)	Root-End Surface Microcracking. Adapted Classification from Rainwater et al. [24] and De Bruyne and De Moor [33].						
	Type					Location	
Designation	Complete	Incomplete				Narrower	Wider
		Intracanal	Extra-canal	Intra-dentinal			
Description	From the root canal to the root surface	Originating from the root canal and radiating into the dentine	Originating from the root surface radiating into the dentine	Confined to the dentine		Located at the narrower side of the remaining dentine surface	Located at the wider side of the remaining dentine surface
**(b)**	**Marginal Integrity** **Adapted Classification from Taschieri et al. [31].**						
**Score**	**0**	**1**		**2**		**3**	
Description	Ideal preparation, with no defects	A single visible defect produced by the contact between the angle of the tip and the cavity margin		Chipped, ragged cavity margin		Chipped, ragged cavity margin plus some defects due to the tips bouncing off the root during root-end preparation	
**(c)**	**Presence of Debris** **Adapted Classification from Khabbaz et al. [34].**						
**Score**	**0**	**1**		**2**	**3**	**4**	
Description	Clean walls	Debris on 1 wall		Debris on 2 walls	Debris on 3 walls	Debris on 4 walls	

**Table 2:** Results of the two experimental groups regarding the number, type, and location of microcracks.

		Immediately after Root-End Resection		T_PO_		T_24H_		T_7D_	
		CVDentus	NSK	CVDentus	NSK	CVDentus	NSK	CVDentus	NSK
Number		0	0	2	2	2	2	2	2
Type	Intracanal	0	0	2	2	2	2	2	2
	Extra-canal	0	0	0	0	0	0	0	0
	Intra-dentinal	0	0	0	0	0	0	0	0
	Complete	0	0	0	0	0	0	0	0
Location	Narrower	0	0	0	0	0	0	0	0
	Wider	0	0	2	2	2	2	2	2

**Table 3:** Frequencies and percentages obtained in the two experimental groups regarding (**a**) marginal integrity and (**b**) presence of debris.

		Frequency		Percentage (%)	
		CVDentus	NSK	CVDentus	NSK
(a) Marginal integrity *	0	4	3	25.0	18.8
	1	10	5	62.5	31.2
	2	2	7	12.5	43.8
	3	0	1	−	6.2
(b) Presence of debris **	0	9	2	56.2	12.5
	1	6	6	37.5	37.5
	2	1	7	6.2	43.8
	3	0	1	−	6.2
	4	0	0	−	−

* N = 32; Mann—Whitney test; *p* = 0.102, ** N = 32; Mann—Whitney test; *p* = 0.003.

**Table 4:** Mean, standard deviation (SD), and minimum and maximum values of each experimental group regarding the time of apical preparation.

	Time of Apical Preparation			
	Mean	SD	Minimum	Maximum
CVDentus	60.56	33.18	30.0	160.0
NSK	55.56	20.41	34.0	96.0

N = 32; Mann—Whitney test; *p* = 0.780.
